# Unveiling a novel function of Aconitase-2: attenuating lung ischemia-reperfusion injury via inhibition of pulmonary endothelial apoptosis

**DOI:** 10.1016/j.redox.2026.104016

**Published:** 2026-01-12

**Authors:** Jiaojiao Sun, Bo Xu, Yijing Chen, Meng Sui, Mochi Wang, Ranming Ma, Jinbo Wu, Shiyong Teng, Qingfeng Pang, Chunxiao Hu

**Affiliations:** a Wuxi School of Medicine, Jiangnan University, 1800 Lihu Avenue, Wuxi, Jiangsu, 214122, China; b Department of Anesthesiology, The Affiliated Wuxi People's Hospital of Nanjing Medical University, Wuxi People's Hospital, Wuxi Medical Center, Nanjing Medical University, 299 Qingyang Road, Wuxi, Jiangsu, 214023, China; c Department of Anesthesiology, First Hospital of Jilin University, 1 Xinmin Street, Changchun, Jilin, 130000, China

**Keywords:** Aconitase-2, Lung ischemia-reperfusion injury, Pulmonary vascular endothelial cells, Mitochondrion, Apoptosis

## Abstract

Mitochondrial dysfunction during lung ischemia-reperfusion injury (LIRI) contributes to organ dysfunction. Aconitase-2 (ACO2), by enhancing the mitochondrial tricarboxylic acid (TCA) cycle in pulmonary vascular endothelial cells (PVECs), plays a critical role in maintaining cellular energy metabolic homeostasis. Single-cell RNA sequencing was performed to characterize cellular phenotypes within the lung tissue microenvironment of I/R mice, and bulk RNA sequencing was applied to identify differentially expressed genes associated with LIRI. Our clinical cohort included 65 healthy donors and 48 patients with LIRI to evaluate the correlation between serum ACO2 levels and lung function. In vivo, using a murine I/R model, we administered an adeno-associated virus for lung-specific ACO2 overexpression, as well as an ACO2 inhibitor (tricarballylic acid), to assess their effects on lung injury. In vitro, primary PVECs were isolated and subjected to hypoxia/reoxygenation (H/R), followed by ACO2 overexpression or knockout, and treatment with the ACO2 downstream metabolite derivative 4-octyl itaconate (4-OI), to investigate its role in mitochondrial function and apoptosis. Serum ACO2 levels were reduced in LIRI patients and exhibited a significant negative correlation with impaired lung function. In I/R mice, ACO2 overexpression ameliorated mitochondrial dysfunction and attenuated lung injury, whereas ACO2 inhibition exacerbated these pathological changes. In PVECs, ACO2 overexpression enhanced mitochondrial function and reduced apoptosis; conversely, ACO2 knockout exerted opposing effects. Notably, supplementation with 4-OI mitigated mitochondrial dysfunction and cellular apoptosis induced by ACO2 deficiency. These findings suggest that ACO2 has therapeutic potential in improving mitochondrial function, reducing apoptosis, and alleviating LIRI, positioning it as a promising target for the treatment of this condition.

## Highlights

• Reduced serum levels of ACO2 are associated with impaired lung function in patients and mice with LIRI.
• ACO2 sustains cellular energy homeostasis in PVECs by enhancing mitochondrial TCA activity.
• Overexpression of ACO2 attenuates LIRI in mice, whereas its inhibition exacerbates pathological outcomes.
• ACO2 improves mitochondrial function and attenuates apoptosis in PVECs.
• The compound 4-OI ameliorates the detrimental effects caused by ACO2 deficiency.

## Graphical abstract

**Figure:**
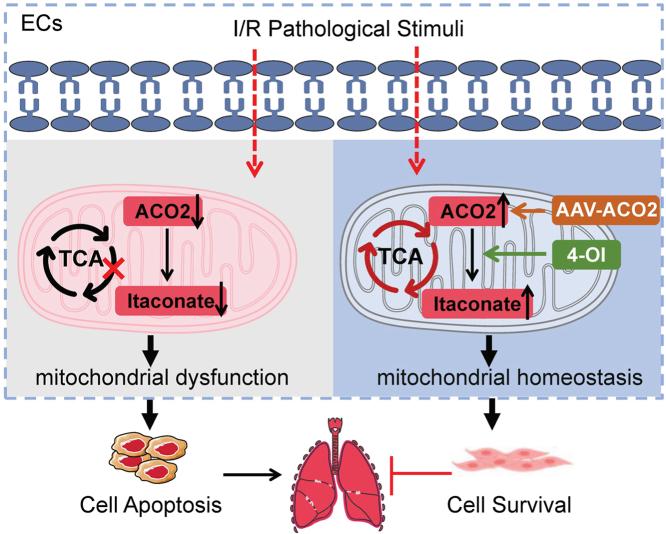

## 1 Introduction

Lung ischemia-reperfusion injury (LIRI) is a critical pathological process that occurs in various clinical settings, including lung transplantation, cardiopulmonary bypass, and trauma [1]. Despite advances in medical management, LIRI continues to contribute significantly to morbidity and mortality, largely due to the complex interplay of oxidative stress, inflammatory responses, and cellular dysfunction during reperfusion [2]. Among the affected cell types, endothelial cells (ECs) are particularly crucial, as they play a central role in maintaining vascular homeostasis and barrier function [3]. Damage to PVECs during LIRI disrupts the endothelial barrier, leading to increased vascular permeability, edema, and ultimately, organ dysfunction [4].

Mitochondria, serving as the central hub of cellular energy metabolism, are profoundly implicated in the pathogenesis of LIRI [5]. During ischemia, oxygen and nutrient deprivation lead to mitochondrial dysfunction, characterized by impaired ATP production and accumulation of metabolic intermediates [6]. Subsequent reperfusion further aggravates mitochondrial injury through a burst of reactive oxygen species (ROS) and disruption of the tricarboxylic acid (TCA) cycle [7]. As a core metabolic pathway in mitochondria, the TCA cycle is essential for ATP generation and maintenance of redox balance. Its dysregulation in PVECs has been increasingly recognized as a key factor in LIRI progression, underscoring the importance of targeting mitochondrial metabolism for therapeutic intervention.

Aconitase-2 (ACO2), a pivotal enzyme in the TCA cycle, catalyzes the conversion of citrate to isocitrate and plays an important role in regulating mitochondrial energy output and redox homeostasis [8]. Growing evidence indicates that ACO2 exerts protective effects in various ischemia-related pathologies by preserving mitochondrial function and alleviating oxidative stress [9 10 11]. However, the specific function of ACO2 in LIRI, particularly within PVECs, remains incompletely understood. Based on the central role of mitochondrial dysfunction in LIRI, we hypothesized that ACO2 may attenuate lung injury by enhancing TCA cycle flux and improving mitochondrial performance in PVECs.

In this study, we aimed to elucidate the role of ACO2 in LIRI and its underlying mechanisms. Using a combination of in vitro and in vivo models, we investigated whether ACO2 targeting could alleviate LIRI by boosting TCA cycle activity and suppressing apoptosis. Our results provide new mechanistic insights into the protective role of ACO2 in LIRI and support its potential as a therapeutic target for reducing lung damage following I/R.

## 2 Materials and methods

### 2.1 Single-cell RNA sequencing (scRNA-seq)

Single-cell suspensions from lung tissue were generated according to a published protocol with slight modifications [12]. Single-cell suspensions were generated from the lung tissues of I/R and sham-operated mice (*n* = 2) via enzymatic digestion (Collagenase IV/DNase I), mechanical dissociation, and filtration. The SeekOne®DD single-cell 3 ′Transcriptome kit includes: microarray (SeekOne®DD Chip S3), Gasket, Carrier Oil, Barcoded Beads, amplification reagent, library construction reagent. Based on the principle of microfluidic technology, the kit can separate and capture single cells through water in oil, and the RNA from different cells is molecularly labeled by nucleic acid modified Barcoded Beads, and sequenced by Illumina, BADA or Truemat high-throughput sequencing platforms. ≧50,000 reads per cell were measured to ensure the accuracy of single-cell sequencing data analysis. Raw data were processed with SeekSoul Online and analyzed in R.

### 2.2 Bulk RNA sequencing (RNA-seq)

Total RNA was extracted from snap-frozen lung tissues of I/R and sham-operated mice (*n* = 6) using TRIzol reagent. RNA quality was verified (RIN >8.0) using an Agilent 2100 Bioanalyzer. Sequencing libraries were prepared from 1 μg of total RNA per sample with the Illumina Stranded mRNA Prep Kit and sequenced on an Illumina NovaSeq 6000 platform (150 bp paired end). Raw reads were processed with Trimmomatic (v0.39) for quality control, aligned to the GRCm39 genome using HiSAT2 (v2.2.1), and counted by featureCounts (v2.0.3) against GENCODE M31. Differential expression analysis was performed with DESeq2 (v1.38.0; FDR <0.05, |log_2_FC| > 1). Functional enrichment of GO and KEGG pathways was conducted using clusterProfiler (v4.6.0).

### 2.3 Mitochondrial metabolomic analysis

Mitochondria were isolated from treated PVECs using a commercial kit, with metabolites extracted in chilled methanol: acetonitrile (1:1). The extracts were analyzed by LC-MS/MS using a HILIC column and a high-resolution mass spectrometer operating in dual-polarity mode. Data processing included peak alignment, metabolite identification via mass matching (HMDB/METLIN), and normalization to mitochondrial protein [13].

### 2.4 Bioinformatic analysis

The transcriptomic dataset GSE127003, comprising lung tissue profiles from a mouse LIRI model, was obtained from the Gene Expression Omnibus (GEO). Data processing and differential expression analysis were conducted in R. Based on the data type, either the limma package or DESeq2 was applied to identify differentially expressed genes (DEGs), defined as those with FDR-adjusted p < 0.05 and |log_2_FC| > 1.

### 2.5 Patients and ethics

Plasma samples were obtained from two distinct cohorts: healthy donors (*n* = 65) and LIRI patients (*n* = 48). The sample collection was conducted at Wuxi People's Hospital Affiliated to Nanjing Medical University from January 1, 2023, to June 30, 2024 (Table S1). A detailed flow chart illustrating the clinical sample collection process was presented in Fig. S1. This study received ethical approval from the Ethics Committee of Wuxi People's Hospital Affiliated with Nanjing Medical University (Ethics Review Number: KY24022).

### 2.6 Reagents and antibodies

Tricarballylic acid (TA; Catalog No. HY-W020215) and 4-octyl itaconate (4-OI; Catalog No. HY-112675) were obtained from MedChem Express (Monmouth Junction, NJ, USA). Endothelial cell-specific medium (Catalog No. 1001) was acquired from ScienCell Research Laboratories (Carlsbad, CA, USA). Detailed information regarding the primary antibodies used in this study was provided in Table S2.

### 2.7 Mouse model of lung ischemia-reperfusion injury and treatment

The animal experiments were approved by the Wuxi People's Hospital Affiliated with Nanjing Medical University (Ethics Approval No. KY24006) and conducted in accordance with established protocols. Pathogen-free male C57BL/6 mice, including wild-type (WT) animals, aged 6–8 weeks, were obtained from the Model Animal Research Center, MARC, Nanjing (No. XM002783). Three weeks prior to I/R surgery, the ACO2 inhibitor TA (20 mg/kg/day) was injected intravenously into mice [14]. WT mice were randomly divided into four experimental groups (*n* = 6): Sham group (Sham), TA-group (TA), I/R group (I/R), and I/R + TA group (I/R + TA). In a separate set of experiments, 4-OI (25 mg/kg/day) was administered intraperitoneally before I/R surgery. WT mice in this cohort were randomly divided into eight groups (n = 6): Sham, 4-OI, TA, 4-OI + TA, I/R, I/R + 4-OI, I/R + TA, and I/R + 4-OI + TA.

Following a cervical incision, the left hilum was exposed. In I/R groups, the left pulmonary vessels and bronchus were occluded with a microvascular clamp for 1 h to induce ischemia, followed by clamp removal to initiate reperfusion (2 h). Sham mice underwent identical surgery without clamping. After reperfusion, the mice were sacrificed, and bronchoalveolar lavage fluid, blood samples, and lung tissue specimens were collected [15].

### 2.8 AAV delivery of ACO2

Three weeks prior to I/R surgery, mice in the AAV-NC and AAV-ACO2 groups received 10 μl of AAV-NC (1.0 × 10^12^ vector genomes [vg]/mL; HanBio, Shanghai, China) or 10 μl of AAV-ACO2 (1.0 × 10^12^ vg/mL; HanBio), respectively, via tail vein injection to achieve lung-specific ACO2 overexpression in I/R mice. Genotyping was performed using the following primers: forward, 5′-GCTAATCGAACTCGAGTTGACTAAAGCCGC ATACGTG.

CTT-3'; reverse, 5′-CGAAACTCGAGTTGACCTGTTCATCGATGAAGTGCTTT.

TG-3' [16].

### 2.9 Blood gas analysis

Arterial blood samples were collected using heparinized syringes from the femoral artery of mice. Blood gas analysis was performed using a blood gas analyzer (the ABL90 FLEX analyzer) capable of measuring parameters including pH, partial pressure of oxygen (PaO_2_), and partial pressure of carbon dioxide (PaCO_2_).

### 2.10 Lung histological assay and wet-to-dry ratio analysis

Mice lung specimens were fixed in 4 % paraformaldehyde for 48h and subsequently embedded in paraffin using ASP200S and EG1150H (Leica). At the time of death, the left lungs of mice were taken and weighed to determine the wet weight, then placed in an oven at 80 °C for 24h and reweighed to determine the dry weight for calculation of the wet/dry weight ratio.

### 2.11 Acquisition and analysis of bronchoalveolar lavage fluid (BALF)

The lungs of mice were lavage three times with 0.8 mL of saline. Subsequently, the BALF was centrifuged at 2000 rpm for 5 min at 4 °C. Following centrifugation, the cells were collected and subjected to cell counting.

### 2.12 Immunohistochemistry staining

Paraffin-embedded lung tissue sections (4 μm) were deparaffinized, rehydrated, and subjected to antigen retrieval in heated citrate buffer. After quenching endogenous peroxidase and blocking, sections were incubated overnight with primary antibody against MPO and subsequently with an HRP-conjugated secondary antibody. Signals were developed with DAB, followed by hematoxylin counterstaining.

### 2.13 Isolation and culture of primary pulmonary vascular endothelial cells

Primary PVECs were isolated according to a well-established protocol [17]. Briefly, perfused lung tissues were minced and digested with 0.1 % collagenase type II at 37 °C for 45 min. The resulting cell suspension was filtered through a 70-μm strainer, and endothelial cells were collected by seeding onto fibronectin-coated dishes. Cells were cultured in endothelial growth medium (EGM-2, Lonza) supplemented with 2 % FBS, VEGF, bFGF, and ECGS at 37 °C with 5 % CO_2_.

### 2.14 Hypoxia/reoxygenation (H/R) model

In the H/R group, cells were subjected to an anoxic environment consisting of 5 % CO_2_, 94 % N_2_, and 1 % O_2_ at 37 °C, followed by reoxygenation in a 95 % air and 5 % CO_2_ atmosphere with 10 % DMEM for an additional 6h [18]. The primary PVECs were categorized into four groups: Vehicel group (Vehicle), TA group (TA), H/R group (H/R) and H/R + TA group (H/R + TA). In TA and H/R + TA group, 5 mM TA was introduced to the culture medium 4h prior to the hypoxia treatment, whereas an equivalent volume of sterile enzyme-free water was added for the Vehicel group. The primary PVECs were categorized into four groups: Vehicel group (Vehicle), 4-OI group (4-OI), H/R group (H/R) and H/R + 4-OI group (H/R + 4-OI). In 4-OI and H/R + 4-OI group, 250 μM 4-OI was introduced to the culture medium 0.5h prior to the hypoxia treatment, whereas an equivalent volume of sterile enzyme-free water was added for the Vehicel group [19].

### 2.15 Biochemical indexes analysis

Contents of MDA (Cat. No: A003-1-2), MPO (Cat. No: A044-1-1), GSH (Cat. No: A006-2-1), T-AOC (Cat. No: A015-2-1), SOD (Cat. No: A001-3-2), and Cyt-c (Cat. No: H190-1-2) were quantified using commercial kits (Nanjing Jiancheng Bioengineering Institute) with a UV-VIS spectrophotometer. Itaconate (MM-92790801) and isocitric acid (#S0523S) concentrations were measured using ELISA (Meimian, Jiangsu, China) and enzymatic (Beyotime, shanghai, China) kits, respectively, following manufacturers' protocols.

### 2.16 Adenosine triphosphate content (ATP) and cell counting kit-8 (CCK-8) assay

Cellular ATP content was quantitatively determined using an enhanced ATP assay kit (#SB-AM1001, Share-Bio, Shanghai, China) according to the manufacturer's protocol. Cell viability was assessed using a CCK-8 assay (#SB-CCK8, Share-Bio, Shanghai, China).

### 2.17 Measurement of electron transport chain (ETC) complex activities

Mitochondrial fractions from cells were resuspended (0.1 μg/μl) in assay buffer. Enzyme activities were spectrophotometrically determined as follows: Complex I by rotenone-sensitive NADH oxidation at 340 nm; Complex II by DCPIP reduction at 600 nm; Complex III by antimycin A-sensitive cytochrome *c* reduction at 550 nm; and Complex IV by cytochrome *c* oxidation at 550 nm [20].

### 2.18 Measurement of metabolic ratios

The ATP/ADP ratio in cells was determined using a bioluminescent assay kit (KA1673, Abnova), with luminescence measured on a Hidex Sense microplate reader. Concurrently, NAD^+^/NADH levels were quantified with a colorimetric assay kit (ab65348, Abcam), and absorbance was read at 450 nm using a Wallac 1420 VICTOR2 multilabel counter.

### 2.19 Measurement of oxygen consumption rate (OCR)

OCR was assessed using a commercial kit (No.600800, Cayman Chemical) [21]. Cells seeded on a 96-well plate were incubated with a phosphorescent oxygen probe and covered with mineral oil. Kinetic readings were obtained at 37 °C over a 2-h period using a microplate reader (BioTek), and basal OCR was calculated from the resulting slope.

### 2.20 JC-1 and MitoSOX red staining

Mitochondrial membrane potential was evaluated in cells using the JC-1 assay (#C2005, Beyotime, Nanjing, China), with fluorescence imaging performed on a fluorescence microscope. Mitochondrial superoxide production was detected by staining cells with MitoSOX Red (#S0061S, Beyotime, Shanghai, China) and imaging with a confocal microscope.

### 2.21 TdT-mediated dUTP nick-end iabeling (TUNEL) staining

TUNEL assays were performed with a one-step TUNEL apoptosis kit (Abbkine, Beijing, China). Paraffin-embedded lung tissue sections and cells were stained according to the manufacturer's protocol.

### 2.22 Transmission electron microscopy

Cells were fixed with 2.5 % glutaraldehyde and post-fixed with 1 % OsO_4_, then stained with uranyl acetate and lead citrate. Ultrastructural images were acquired using a Hitachi H-7650 transmission electron microscope.

### 2.23 Reverse transcription quantitative polymerase chain reaction (RT-qPCR)

Total RNA from cells or tissues was extracted with trizol reagent and reverse transcribed into cDNA. Relative gene expression was calculated by the 2^−ΔΔCt^ method with GAPDH as the endogenous control. Primer sequences were listed in Table S3.

### 2.24 Western blot

Proteins were extracted from tissues or cells using RIPA lysis buffer. Protein bands were visualized using a chemiluminescent detection system (Bio-Rad).

### 2.25 Constructing stable ACO2-knockout cells using a CRISPR/Cas9 system

To generate ACO2-knockout (ACO2-KO) HUVECs, we used CRISPR/Cas9 system as described elsewhere [22]. Cells were seeded in a 6-well culture plate and subsequently transfected when reaching 60–70 % confluence. Thereafter, ACO2-CRISPR/Cas9-KO plasmid (sc-406429, 2 μg) and ACO2 homology-directed DNA repair (HDR) plasmid (2 μg) were mixed with 7 μl of lipofectamine 3000 and 5 μl of P 3000 reagent (Invitrogen, Waltham, MA) and co-transfected into HUVECs. Puromycin was added for 5-7d to select positively transfected cells.

### 2.26 Plasmid construction and transfection

The ACO2 overexpression plasmid, pcDNA3.1(+)-ACO2-3 × FLAG-P2A-EGFP (forward primer [CMV-F]: AATGGGCGCCGTAGGCGTG; reverse primer [EGFP-SEQR]: TGAACTCGCTGAATCTTGTGGC), along with the control vector H2713 pcDNA3.1(+)-3 × FLAG-P2A-EGFP, were designed and constructed by OBiO Technology. HEK-293T cells were plated at a density of 5 × 10^5 cells per well in six-well plates and transfected with 2 μg of plasmid DNA per well using 5 μl of Liposomal Transfection Reagent (Yeasen; Cat. No: 40802ES02), following the manufacturer's protocol.

### 2.27 Dual-luciferase reporter assay

HEK-293T cells were plated in 96-well plates at a density of 5 × 10^3^ cells per well and transfected with either the wild-type (ACO2-wt) or mutant (ACO2-mut) luciferase reporter plasmids. The ACO2-wt plasmid was constructed using the forward primer [RVprimer3] (GCAAAATAGGGCAAGCTGTCCC) and reverse primer [Luc2-N-Re] (AGTGGCGTTCGGTAGAATG), while the ACO2-mut plasmid was generated with the forward primer [RVprimer3] (TTCTGCCCCCCATCGTGATACC) and reverse primer [Luc2-N-Re] (TGTCCAGCAGCGGCTG). After 48h of transfection, luciferase activity was measured using a dual luciferase reporter assay system (Yeasen, Biotechnology, China).

### 2.28 Electrophoretic mobility shift assays (EMSA)

GLRY1 protein was extracted from cell lysates by centrifugation. Synthesized DNA probes targeting GLRY1-ACO2 binding sequences were incubated with the protein extract in EMSA buffer. Protein-DNA complexes were then separated from a native polyacrylamide gel.

### 2.29 Statistical analysis

Data are presented as mean ± SD from at least three independent experiments. Group comparisons were analyzed by one-way ANOVA, while differences between LIRI patients and healthy controls were assessed using Student's t-test or Mann-Whitney *U* test, as appropriate. Correlation analysis was performed using linear regression. A *P* -value <0.05 was considered statistically significant. All analyses were conducted using GraphPad Prism v9.0 and IBM SPSS v20.

## 3 Results

### 3.1 Single-cell and transcriptomic profiling of endothelial cells in I/R mice

**Fig. 1:**
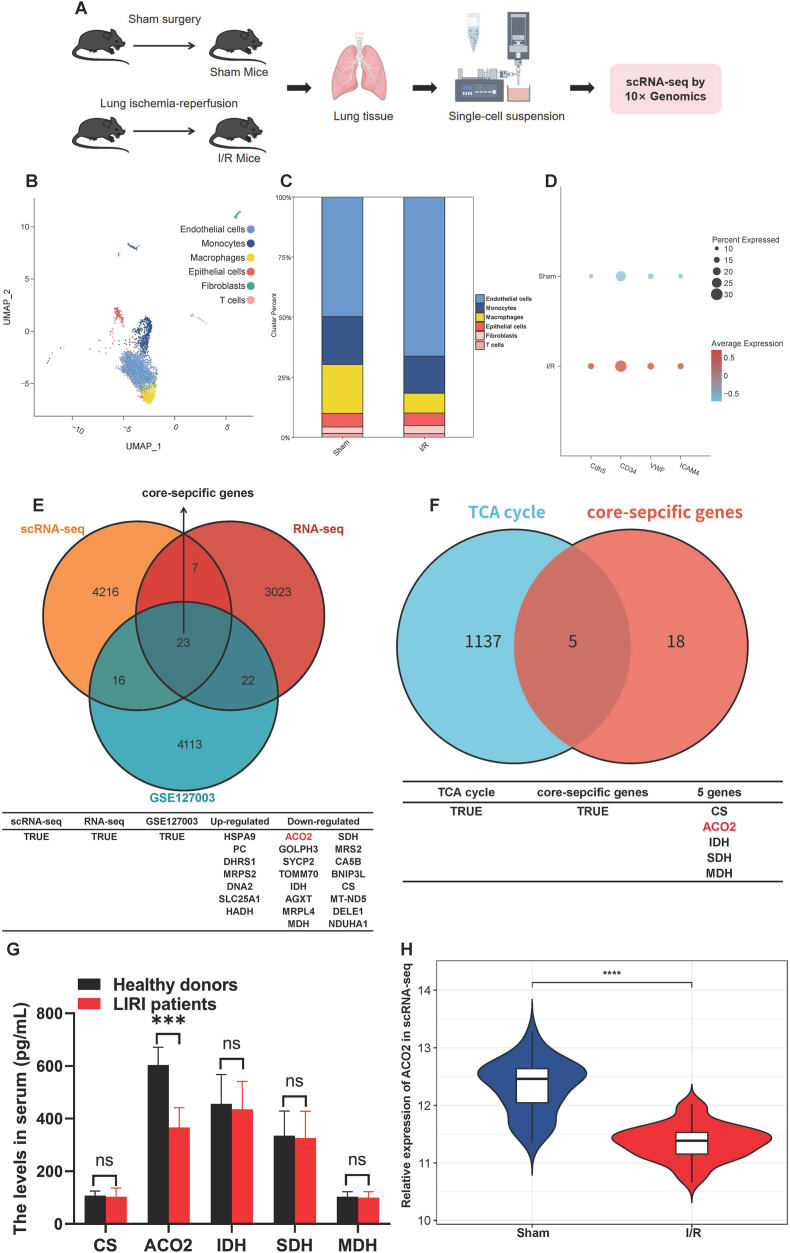
**Characterization of the endothelial cell transcriptome in I/R mice using single-cell RNA s**equencing. (A) Schematic diagram illustrating the workflow of sample preparation, scRNA-seq, and bioinformatic analysis. (B) The t-SNE plot representing 12,449 cells partitioned into 6 distinct clusters, with each dot indicating an individual cell colored by cluster identity. (C) Histogram indicated the proportion of cells in the lung tissue for both analyzed samples. (D) Expression patterns of established endothelial cell markers (Cdh5, CD34, VWF, and ICAM4) under LIRI conditions. (E) Venn diagram of hub genes between DEGs from the scRNA-seq, bulk RNA-seq, and GSE127003. (F) Venn diagram showing the intersection between TCA cycle-related genes and the 23 core-specific genes. (G) Serum contents of CS, ACO2, IDH, SDH, and MDH measured by ELISA in healthy donors and LIRI patients. (H) The ACO2 expression in the Sham and I/R group. Data are presented as the mean ± SD; LIRI patients (*n* = 48) and healthy donors (*n* = 65); ∗∗∗*P* < 0.001, and ns indicates no significant difference.

To identify key cellular populations involved in I/R mice, we performed scRNA-seq on 2,1522 cells from mouse lung tissues using the 10 × Genomics platform. After quality control, 12,449 high-quality cells were retained and clustered into 6 distinct subsets (Fig. 1A and B). Fibroblasts and endothelial cells constituted the most abundant populations, with increased proportions of Endothelial cells, Monocytes, Macrophages, Epithelial cells, Fibroblasts, and T cells in I/R tissues (Fig. 1C). Expression of endothelial markers (Cdh5, CD34, VWF, ICAM4) was elevated post-I/R (Fig. 1D). KEGG analysis of scRNA-seq and RNA-seq data revealed upregulation of the TCA cycle in I/R samples (Fig. S2A and B). Integration of DEGs from scRNA-seq, bulk RNA-seq, and dataset GSE127003 identified 23 core-specific genes (Fig. 1E). Intersection with TCA cycle-related genes yielded 5 candidate hub genes (Fig. 1F). Serum ACO2 levels decreased in LIRI patients (Fig. 1G), and ACO2 expression was downregulated in mouse lung tissue following I/R (Fig. 1H). These findings indicated that ACO2 was significantly decreased in LIRI and underscore the pivotal role of ECs in I/R pathogenesis.

### 3.2 ACO2 decreased in LIRI patients, I/R mice, and H/R-primary PVECs

**Fig. 2:**
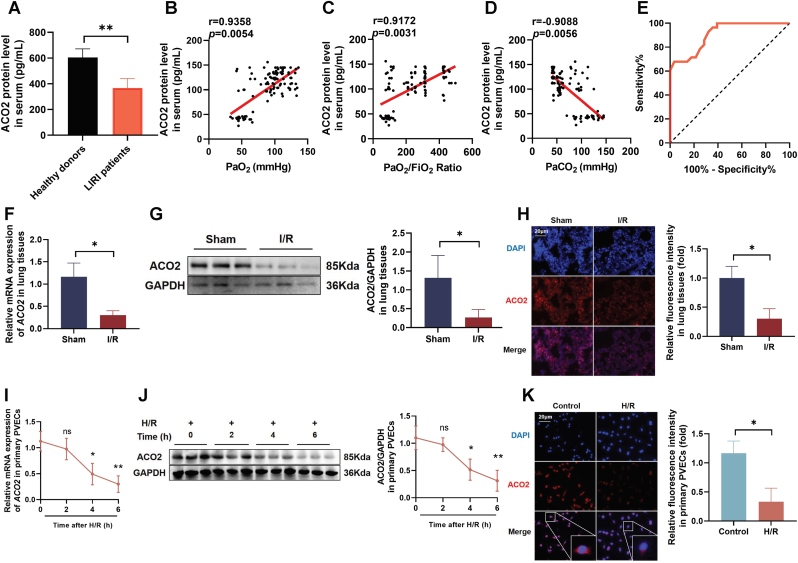
**Decreased expression of ACO2 in LIRI patients, I/R mice, and H/R-treated prim**ary PVECs. (A) Serum ACO2 contents measured by ELISA in LIRI patients and healthy donors. (B–D) Correlation analyses between serum ACO2 contents and pulmonary function parameters: (B) PaO_2_, (C) PaO_2_/FiO_2_ ratio, and (D) PaCO_2_ in LIRI patients. (E) ROC curve evaluating the diagnostic value of serum ACO2 for LIRI. (F) *ACO2* mRNA levels in lung tissues of I/R and sham mice, determined by RT-qPCR (*n* = 6). (G) Representative western blot and quantification of ACO2 protein contents in lung tissues (*n* = 6). (H) Immunohistochemical staining and semi-quantitative analysis of ACO2 in mouse lung sections (scale bar = 20 μm). (I) *ACO2* mRNA expression in primary PVECs under H/R, assessed by RT-qPCR. (J) Western blot and quantification of ACO2 protein contents in primary PVECs after H/R. (K) Immunofluorescence staining of ACO2 (red) and DAPI (blue) in primary PVECs (scale bar = 20 μm). Data are presented as the mean ± SD; LIRI patients (*n* = 48) and healthy donors (*n* = 65) (A–E); *n* = 6 (F–H); *n* = 3 (I–K); ∗*P* < 0.05, ∗∗*P* < 0.01, and ns indicates no significant difference.

To investigate ACO2 expression dynamics in LIRI, we first analyzed serum ACO2 contents in a clinical cohort (65 healthy donors and 48 LIRI patients; Table S1). ELISA revealed significantly decreased serum ACO2 in LIRI patients (Fig. 2A), which correlated positively with PaO_2_ (Fig. 2B) and PaO_2_/FiO_2_ ratio (Fig. 2C), and negatively with PaCO_2_ (Fig. 2D). ROC analysis demonstrated high diagnostic accuracy for LIRI (AUC = 0.9088, Fig. 2E). In LIRI models, ACO2 expressions were significantly downregulated at both mRNA and protein contents in lung tissues (Fig. 2F–H). Consistently, primary PVECs subjected to H/R showed reduced ACO2 expression (Fig. 2I–K). These findings indicated that ACO2 was significantly suppressed in LIRI patients, I/R mice and H/R-stimulated PVECs.

### 3.3 ACO2 regulated mitochondrial metabolism through the TCA cycle in primary PVECs

**Fig. 3:**
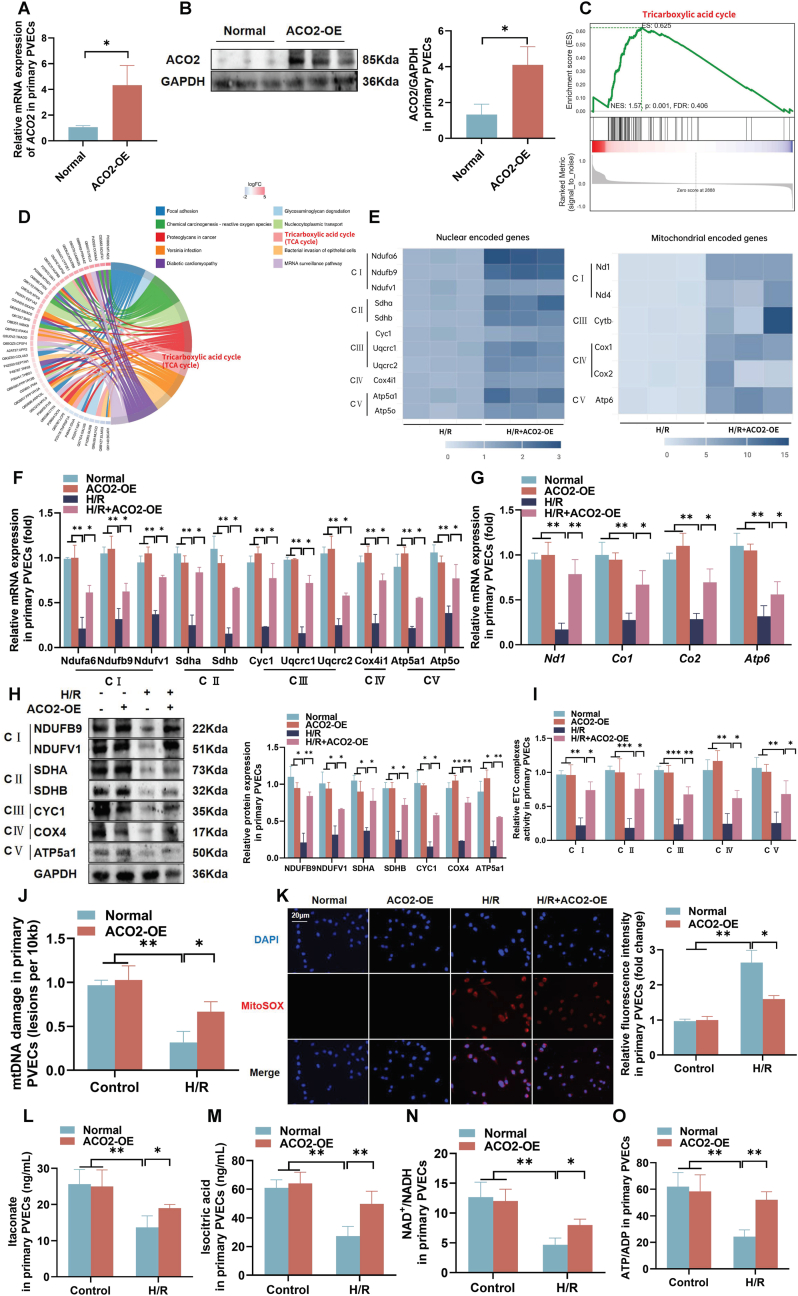
**ACO2 regulated mitochondrial metabolism through the TCA cycle in primary PVECs.** (A) The RT-qPCR analysis of *ACO2* mRNA expression in primary PVECs. (B) Western blot and quantitative analysis of ACO2 protein contents. (C) GSEA enrichment plot illustrating ACO2-activated signaling pathways. (D) KEGG pathway analysis of DEGs. (E) Heatmap displaying expression changes (log_2_ fold-change) of nuclear- and mitochondrial-encoded genes involved in mitochondrial function. (F–G) The mRNA expression levels of nuclear-encoded and mtDNA-encoded mitochondrial genes. (H) The protein contents of ETC complex subunits. (I) Activities of mitochondrial ETC complexes. (J) Assessment of mtDNA damage. (K) Mitochondrial reactive oxygen species levels detected by MitoSOX Red staining (scale bar = 20 μm). (L–M) Metabolomic analysis of TCA cycle intermediates: itaconate and isocitric acid. (N–O) Metabolic ratios of NAD^+^/NADH and ATP/ADP. Data are presented as the mean ± SD; *n* = 3; ∗*P* < 0.05, ∗∗*P* < 0.01, and ∗∗∗*P* < 0.001.

We examined the protective potential of ACO2 overexpression (ACO2-OE) against H/R-induced injury in primary PVECs. Verification by RT-qPCR and western blot confirmed successful ACO2-OE at both transcriptional and protein contents (Fig. 3A and B). To explore the mechanistic basis, mitochondrial metabolomic profiling was performed, with bioinformatic analysis revealing significant enrichment of upregulated genes in metabolic pathways, particularly the TCA cycle (Fig. 3C and D). Further studies showed that H/R reduced the expression of nuclear encoded genes and mtDNA encoded genes associated with ETC complexes, whereas ACO2-OE enhanced the expression of these genes (Fig. 3E), which was confirmed by RT-qPCR and western blot experiments (Fig. 3F–H). Functionally, H/R led to a decrease in ETC complex activity and ACO2-OE increased ETC complex activity (Fig. 3I). H/R increased mtDNA damage and caused attenuation of MitoSOX signaling, while ACEO2-OE reduced mtDNA damage and increased MitoSOX signaling (Fig. 3J and K). Metabolomic analysis also revealed that ACO2-OE elevated key TCA cycle intermediates, including itaconate and isocitric acid (Fig. 3L and M), and increased both NAD^+^/NADH and ATP/ADP ratios (Fig. 3N and O). Together, these results demonstrated that ACO2-OE sustained mitochondrial metabolic and redox homeostasis, highlighting its central role in counteracting H/R-induced mitochondrial dysfunction.

### 3.4 ACO2 overexpression attenuated lung injury in I/R mice

**Fig. 4:**
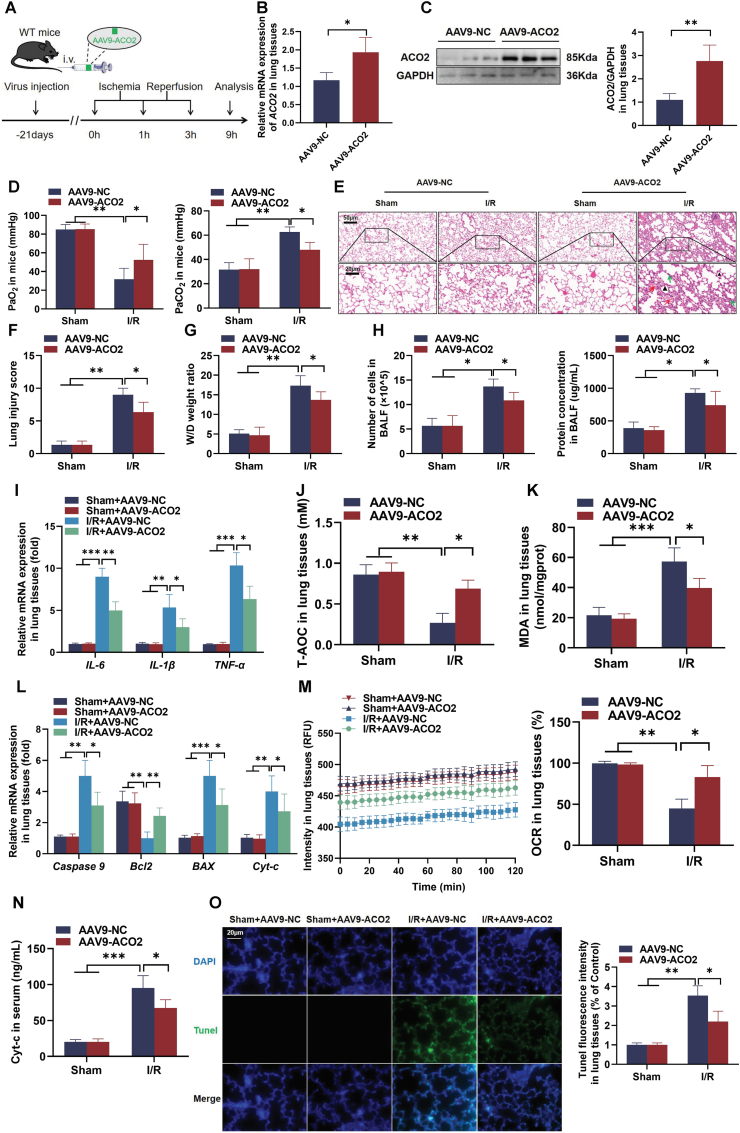
**ACO2 overexpression protected against LIRI in mice. (A) Schematic of AAV9-mediated ACO2-OE in I/R mice.** (B–C) *ACO2* expression levels assessed by RT-qPCR and western blot. (D) Blood gas parameters (PaO_2_ and PaCO_2_) following I/R injury. (E) Representative H&E-stained lung sections (scale bars = 20/50 μm, Black arrow: Alveolar expansion; Red arrow: Hemorrhage; Green arrow: Alveolar septum thickening and inflammatory cell infiltration). (F–G) Quantitative assessment of lung injury via histopathological scoring and wet/dry weight ratio. (H) Total cells count and protein concentration in BALF. (I) The mRNA expression of pro-inflammatory cytokines (*IL-6*, *IL-1β*, and *TNF-α*). (J–K) Oxidative stress markers: T-AOC and MDA. (L) Apoptosis-related gene expression (*caspase 9*, *Bcl-2, BAX*, and *Cyt-c*). (M) Mitochondrial respiratory function measured by OCR. (N) Serum Cyt-c release. (O) TUNEL staining for apoptosis detection (scale bar = 20 μm). Data are presented as the mean ± SD; *n* = 6; ∗*P* < 0.05, ∗∗*P* < 0.01, and ∗∗∗*P* < 0.001.

To evaluate the lung effect of ACO2 against I/R in mice, we applied AAV9 to overexpress ACO2 (AAV9-ACO2) in lungs, and a I/R model was constructed (Fig. 4A). ACO2 overexpression induced by AAV9 delivery was confirmed via RT-qPCR and western blot (Fig. 4B and C). Blood gas analysis revealed that I/R significantly decreased PaO_2_ and increased PaCO_2_ levels compared to the sham group, both of which were mitigated by AAV9-ACO2 (Fig. 4D). Histopathological examinations showed that ACO2 overexpression attenuated I/R-induced alveolar wall thickening (Fig. 4E), accompanied by reduced lung injury scores (Fig. 4F) and decreased wet/dry weight ratios (Fig. 4G). In BALF, AAV9-ACO2 significantly lowered total cell counts and protein concentration (Fig. 4H), as well as pro-inflammatory cytokine levels (*IL-6*, *IL-1β*, and *TNF-α*) (Fig. 4I). Furthermore, AAV9-ACO2 increased T-AOC and reduced MDA contents (Fig. 4J and K). At the molecular level, AAV9-ACO2 downregulated pro-apoptotic genes (*caspase 9*, *BAX*, and *Cyt-c*) and upregulated the anti-apoptotic gene *Bcl-2* (Fig. 4L). In AAV9-ACO2 mice, the oxygen consumption rate (OCR) was also restored (Fig. 4M). Consistent with this, serum cytochrome *c* release was reduced (Fig. 4N), and the TUNEL staining confirmed the suppression of I/R-induced apoptosis following AAV9-ACO2 (Fig. 4O). Collectively, these results demonstrated that AAV9-ACO2 attenuated lung injury in I/R mice.

### 3.5 ACO2-KO and TA exacerbated mitochondrial damage and apoptosis in HUVECs

**Fig. 5:**
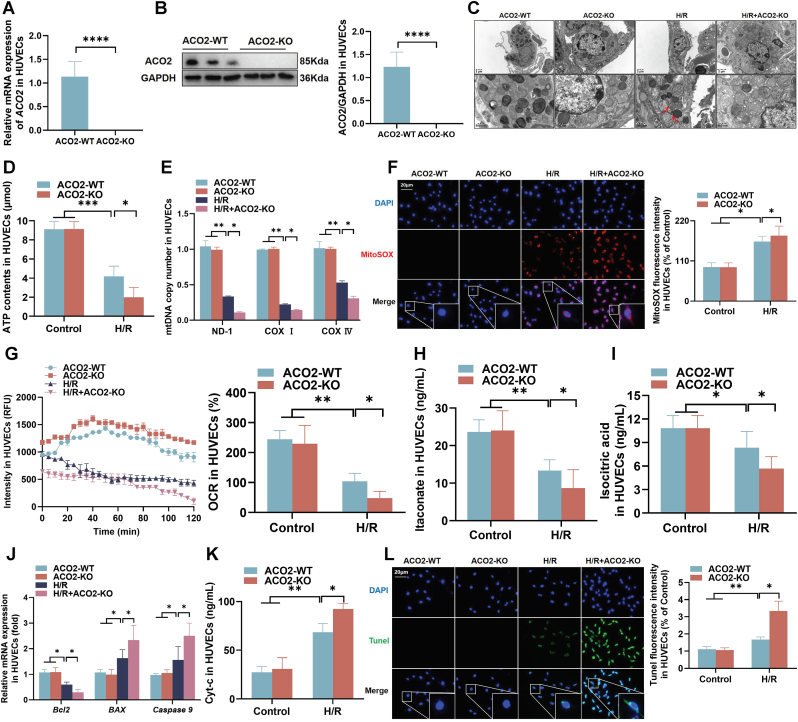
**ACO2-KO exacerbated mitochondrial damage and apoptosis** in HUVECs. (A–B) Validation of ACO2-KO efficiency by RT-qPCR and western blot. (C) Representative TEM images showing mitochondrial ultrastructural changes. The red arrows indicate the disappearance of mitochondrial cristae. (D) Cellular ATP contents measured by enzymatic assay. (E) The mtDNA copy number assessed via quantification of mitochondrial genes (ND-1, COX I, and COX IV). (F) Mitochondrial superoxide production detected by MitoSOX Red staining (scale bar = 20 μm). (G) Mitochondrial respiratory function measured by OCR. (H–I) Metabolomic analysis of TCA cycle intermediates: itaconate and isocitrate. (J) The mRNA expression of apoptosis-related genes (*Bcl-2, BAX,* and *Caspase-9*). (K) Cyt-c release from mitochondria. (L) Apoptosis evaluation by TUNEL staining (scale bar = 20 μm). Data are presented as the mean ± SD; *n* = 3; ∗*P* < 0.05, ∗∗*P* < 0.01, ∗∗∗*P* < 0.001, and ∗∗∗∗*P* < 0.0001.

To further investigate the role of ACO2 in HUVECs following H/R injury, we established an ACO2-KO model. The RT-qPCR and western blot analysis confirmed significant reduction of ACO2 at both mRNA and protein contents (Fig. 5A and B). Transmission electron microscopy revealed that ACO2-KO aggravated mitochondrial ultrastructural damage (Fig. 5C). ACO2-KO led to decreased ATP production and reduced mtDNA copy number (Fig. 5D and E). MitoSOX Red fluorescence intensity, indicative of mitochondrial superoxide levels, was elevated in H/R-HUVECs and further enhanced by ACO2-KO (Fig. 5F). The OCR was also impaired in ACO2-KO HUVECs (Fig. 5G). Metabolically, ACO2-KO suppressed itaconate production (Fig. 5H and I). At the molecular level, ACO2-KO downregulated the mRNA levels *Bcl-2* and upregulated *BAX* and *caspase-9*, accompanied by increased Cyt-c release (Fig. 5J and K). TUNEL staining further confirmed that ACO2-KO exacerbated H/R-induced apoptosis in HUVECs (Fig. 5L). Together, these results demonstrated that ACO2-KO aggravated mitochondrial dysfunction and promoted apoptosis in HUVECs under H/R stress.

**Fig S3:**
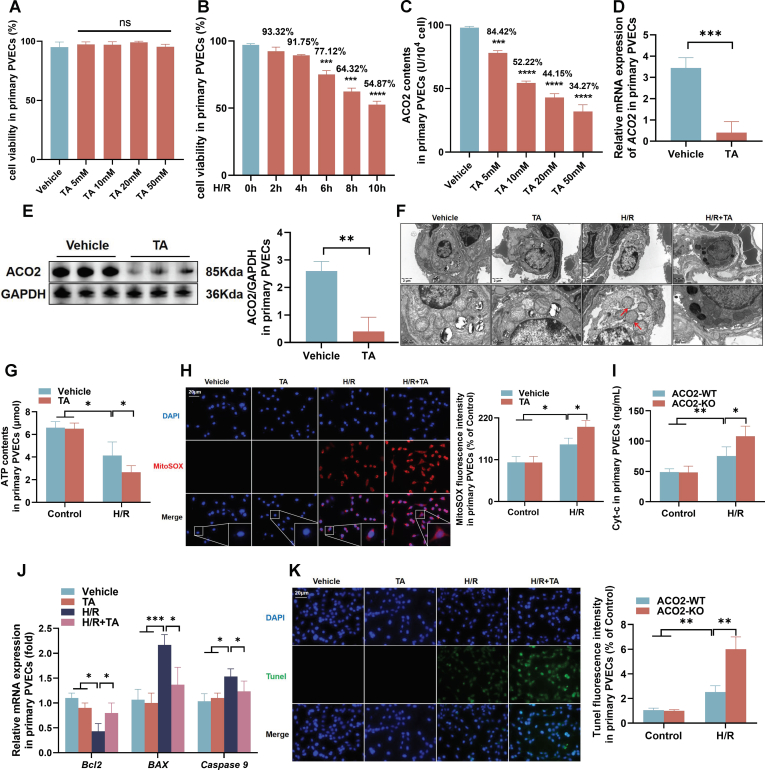


To further investigate the functional consequences of ACO2 suppression, we employed pharmacological inhibition using TA (ACO2 Inhibitor) in primary PVECs. Initial assessment indicated that TA treatment did not significantly affect cell viability under baseline conditions (Fig. S3A). However, TA administration markedly exacerbated cellular injury following 6h of H/R exposure (Fig. S3B). Dose-response analysis identified 5 mM TA as the optimal concentration for effective ACO2 inhibition (Fig. S3C). Subsequent RT-qPCR and western blot analyses confirmed that TA treatment significantly reduced both mRNA and protein expression contents of ACO2 (Fig. S3D and E). TEM revealed that ACO2 inhibition aggravated mitochondrial ultrastructural damage (Fig. S3F). Functional assessment demonstrated that TA treatment reduced ATP production (Fig. S3G) and enhanced mitochondrial superoxide generation, as indicated by increased MitoSOX Red fluorescence intensity following H/R (Fig. S3H). Mechanistic investigations showed that ACO2 inhibition promoted Cyt-c release and dysregulated apoptosis-related gene expression, characterized by upregulation of *Bcl-2* and downregulation of *BAX* and *caspase-9* (Fig. S3I and J). TUNEL staining further confirmed that ACO2 inhibition significantly exacerbated H/R-induced apoptosis in primary PVECs (Fig. S3K). Collectively, these findings demonstrated that pharmacological inhibition of ACO2 aggravated mitochondrial dysfunction and promoted apoptosis under H/R conditions.

**Fig S4:**
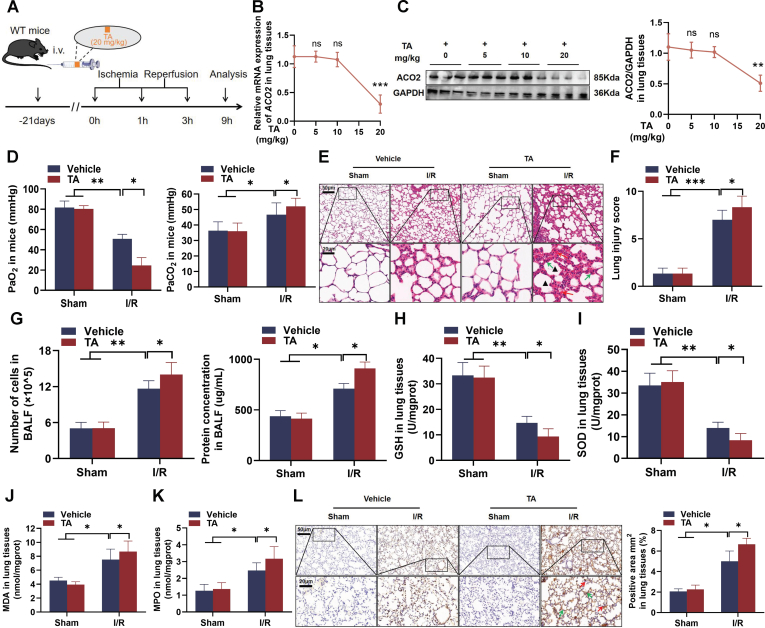


To further determine whether ACO2 inhibitor exerts similar effects in I/R mice, we administered the ACO2 inhibitor TA to I/R mice (Fig. S4A). Based on RT-qPCR and western blot analyses, a dose of 20 mg/kg TA was selected for effective ACO2 suppression in lung tissue (Fig. S4B and C). Blood gas analysis revealed that TA treatment significantly reduced PaO_2_ and increased PaCO_2_ levels in I/R mice (Fig. S4D). Histological examination showed marked alveolar wall thickening following TA administration (Fig. S4E), accompanied by elevated lung injury scores (Fig. S4F). TA also increased total cell counts and protein concentration in BALF (Fig. S4G), indicating enhanced vascular permeability and inflammatory cell infiltration. Oxidative stress evaluation demonstrated that TA further decreased the activities of SOD and GSH (Fig. S4H and I), while increasing contents of MDA and MPO (Fig. S4J and K). Immunohistochemical analysis confirmed enhanced MPO activity in lung tissues after TA treatment (Fig. S4L). Collectively, these findings indicated that pharmacological inhibition of ACO2 aggravated pulmonary injury, oxidative stress, and inflammatory responses in I/R mice.

### 3.6 4-OI rescued mitochondrial dysfunction after ACO2-KO in HUVECs

**Fig S5:**
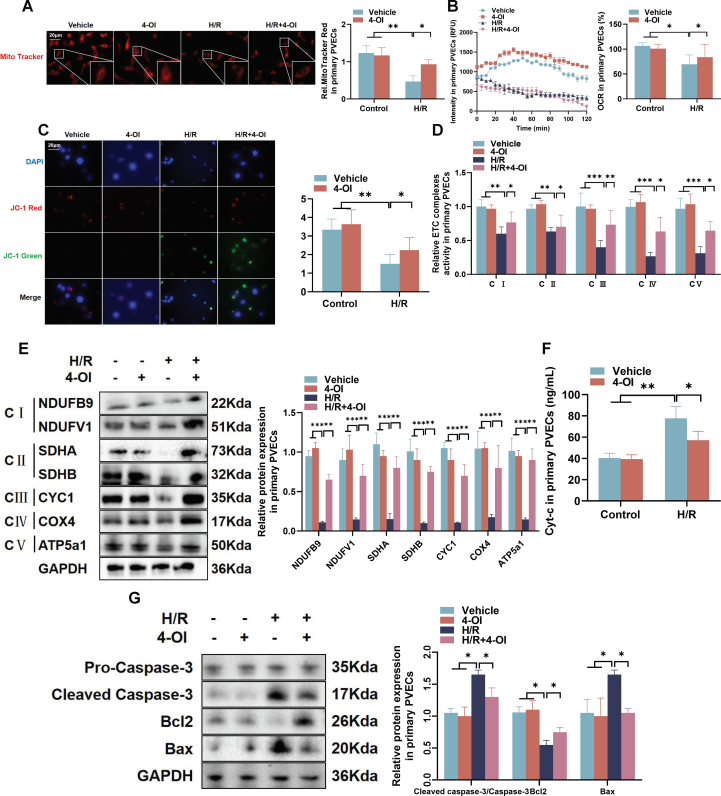


Within the TCA cycle, ACO2 catalyzes the conversion of citrate to itaconate. Given the observed impairment of TCA cycle function in primary PVECs under H/R stress, we investigated whether supplementation with itaconate, a key downstream metabolite, could rescue this dysfunction. Due to the limited membrane permeability of itaconate-a polar α, β-unsaturated dicarboxylic acid-we utilized its cell-permeable derivative, 4-OI, to facilitate cellular uptake and functional analysis. Our results demonstrated that H/R significantly attenuated MitoTracker fluorescence, indicating impaired mitochondrial content, while 4-OI treatment restored and enhanced this signal (Fig. S5A). Concurrently, 4-OI improved mitochondrial respiration, as reflected by increased oxygen consumption rates (Fig. S5B). Furthermore, 4-OI reduced the JC-1 aggregate (red fluorescence) to monomer (green fluorescence) ratio, suggesting stabilization of mitochondrial membrane potential (Fig. S5C). Treatment with 4-OI also enhanced the activity of mitochondrial electron transport chain complexes (Fig. S5D), increased the protein contents of complex subunits, and reduced Cyt-c release (Fig. S5E–F). Importantly, 4-OI exerted anti-apoptotic effects, as evidenced by decreased protein contents of cleaved caspase-3, Bcl-2, and BAX (Fig. S5G). In summary, these findings indicated that supplementation with 4-OI, effectively alleviated H/R-induced mitochondrial dysfunction and cellular injury in HUVECs, highlighting its potential therapeutic role in maintaining mitochondrial homeostasis under pathological conditions.

**Fig. 6:**
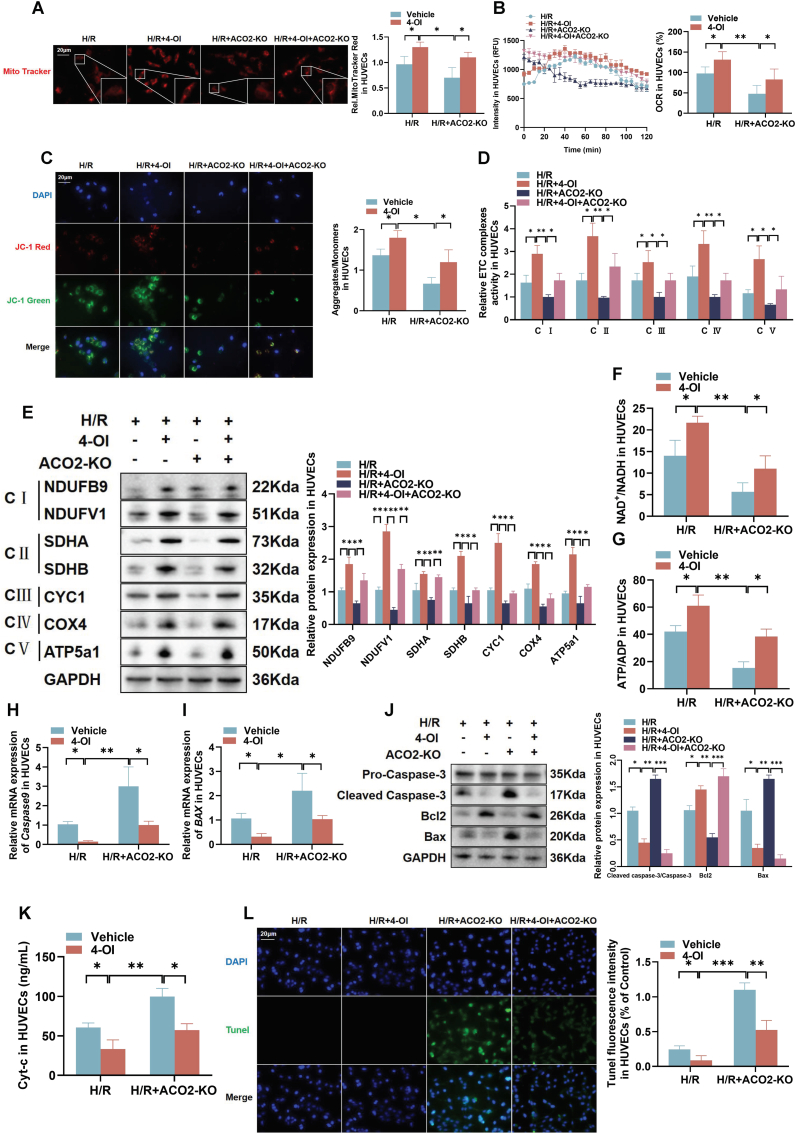
**4-OI rescued mitochondrial dysfunction after ACO2-KO** in HUVECs. (A) Mitochondrial mass assessed by MitoTracker staining (scale bar = 20 μm). (B) Mitochondrial respiratory function measured by OCR. (C) Mitochondrial membrane potential evaluated using JC-1 staining (scale bar = 20 μm). (D) Activities of mitochondrial ETC complexes. (E) Protein contents of ETC complex subunits. (F–G) Metabolic ratios of NAD^+^/NADH and ATP/ADP. (H–I) The mRNA expression of apoptosis-related genes (*caspase-9* and *BAX*). (J) Apoptosis-related protein contents (cleaved caspase-3, Bcl-2, and BAX) analyzed by western blot with semi-quantitative analysis. (K) Cyt-c release from mitochondria. (L) Apoptosis evaluation by TUNEL staining (scale bar = 20 μm). Data are presented as the mean ± SD; *n* = 3; ∗*P* < 0.05, ∗∗*P* < 0.01, and ∗∗∗*P* < 0.001.

We next investigated whether supplementation with 4-OI could mitigate cellular injury following ACO2-KO in HUVECs. We observed that H/R significantly reduced MitoTracker fluorescence, indicating loss of mitochondrial integrity, while 4-OI treatment effectively restored this signal (Fig. 6A). Measurement of OCR demonstrated that 4-OI improved mitochondrial respiration after ACO2-KO (Fig. 6B). 4-OI treatment decreased the JC-1 aggregate-to-monomer ratio, reflecting stabilization of mitochondrial membrane potential (Fig. 6C). Although ACO2-KO impaired mitochondrial complex activity, 4-OI administration restored this function (Fig. 6D) and increased the protein contents of electron transport chain complex subunits (Fig. 6E). Furthermore, 4-OI elevated both NAD^+^/NADH and ATP/ADP ratios (Fig. 6F and G), indicating improved energy metabolism. At the molecular level, 4-OI downregulated mRNA expression of *caspase 9* and *BAX* (Fig. 6H–I). Importantly, 4-OI reduced protein contents of cleaved caspase-3, Bcl-2, and BAX, while decreasing Cyt-c release (Fig. 6J and K), demonstrating potent anti-apoptotic effects. TUNEL staining confirmed that 4-OI attenuated H/R-induced apoptosis after ACO2-KO (Fig. 6L). These findings provided compelling evidence that 4-OI supplementation effectively counteracted ACO2-KO-induced cellular damage, highlighting its therapeutic potential in preserving mitochondrial function and cell viability under pathological stress.

### 3.7 4-OI supplementation attenuated ACO2 inhibition-associated lung injury in I/R mice

**Fig. 7:**
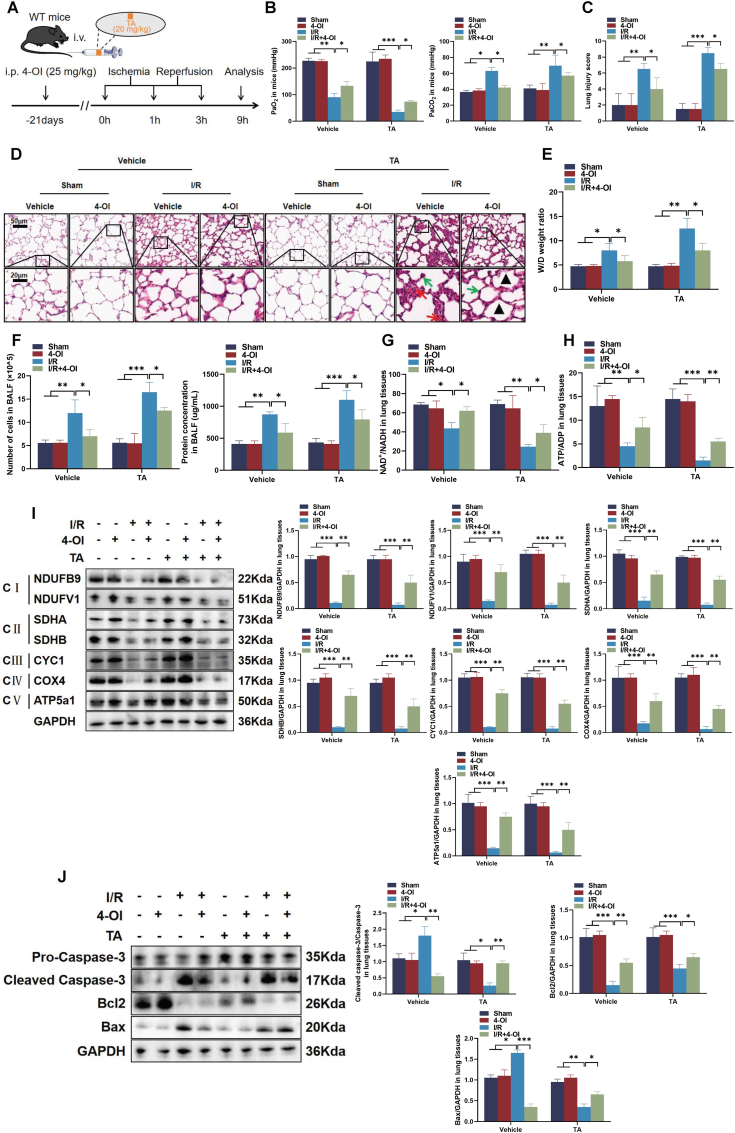
**4-OI supplementation attenuated the lung injury caused by ACO2 inhibition in I/R mice.** (A) Experimental schematic of TA and 4-OI administration in I/R mice. (B) Blood gas parameters (PaO_2_ and PaCO_2_) following I/R injury. (C) Quantitative assessment of lung injury via histopathological scoring. (D) Representative H&E-stained lung sections (scale bars = 20/50 μm, Black arrow: Alveolar expansion; Red arrow: Hemorrhage; Green arrow: Alveolar septum thickening and inflammatory cell infiltration). (E) Pulmonary edema assessment by wet/dry weight ratio. (F) Total cells count and protein concentration in BALF. (G–H) Metabolic ratios of NAD^+^/NADH and ATP/ADP. (I) Protein contents of ETC complex subunits. (J) Apoptosis-related protein contents (cleaved caspase-3, Bcl-2, and BAX) analyzed by western blot with semi-quantitative analysis. Data are presented as the mean ± SD; *n* = 6; ∗*P* < 0.05, ∗∗*P* < 0.01, and ∗∗∗*P* < 0.001.

We evaluated the effects of 4-OI and TA in the model of I/R injury (Fig. 7A). Blood gas analysis showed that 4-OI alleviated the TA-induced reduction in PaO_2_ and elevation in PaCO_2_ (Fig. 7B). 4-OI also reduced the TA-associated increase in lung injury scores (Fig. 7C). Compared to sham controls, 4-OI significantly attenuated TA-induced alveolar wall thickening and elevation in lung wet/dry weight ratios (Fig. 7D and E). Furthermore, 4-OI decreased the TA-mediated increases in total cell counts and protein concentration in BALF (Fig. 7F). 4-OI also reversed the TA-induced reductions in NAD^+^/NADH and ATP/ADP ratios (Fig. 7G and H) and restored mitochondrial complex protein contents (Fig. 7I). At the molecular level, 4-OI exerted anti-apoptotic effects by upregulating Bcl-2 and downregulating cleaved caspase-3 and BAX contents (Fig. 7J). These results provided strong evidence that 4-OI supplementation effectively mitigated lung injury induced by ACO2 inhibition in a I/R mouse model, highlighting its potential as a therapeutic agent for ameliorating mitochondrial dysfunction and cellular damage under ischemic conditions.

### 3.8 GLYR1 bound the ACO2 promoter and enhances its transcriptional activity in HEK-293T cells

**Fig. 8:**
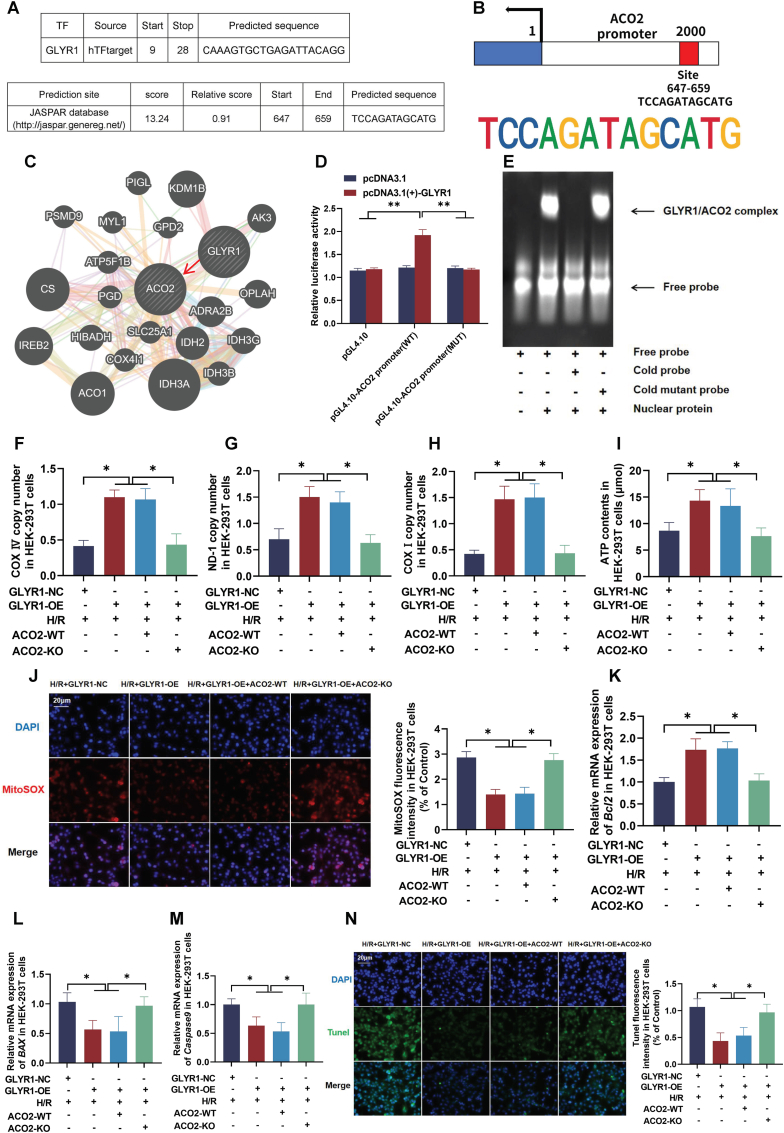
**GLYR1 bound the ACO2 promoter and enhances its transcriptional activity in HEK-2**93T cells. (A) Prediction of ACO2 upstream transcriptional regulators using HumanTFDB. (B) Putative GLYR1 binding motifs in the ACO2 promoter region from JASPAR. (C) Physical interaction between GLYR1 and ACO2 promoters via HumanBase analysis. (D) Dual-luciferase reporter assay evaluating GLYR1 binding to the ACO2 promoter, normalized to co-transfected pRL-TK. (E) EMSA demonstrating specific GLYR1-ACO2 promoter interaction; arrows indicate protein-DNA complexes. (F–H) Mitochondrial electron transport chain complex activities. (I) Cellular ATP content measured by enzymatic assay. (J) Mitochondrial superoxide levels detected by MitoSOX Red staining (scale bar = 20 μm). (K–M) The mRNA expression of apoptosis-related genes (*Bcl-2, BAX,* and *caspase-9*) by RT-qPCR. (N) Apoptosis evaluation by TUNEL staining (scale bar = 20 μm). Data are presented as the mean ± SD; *n* = 3. *P* values were calculated using one-way ANOVA; ∗*P* < 0.05, and ∗∗*P* < 0.01.

**Fig S6:**
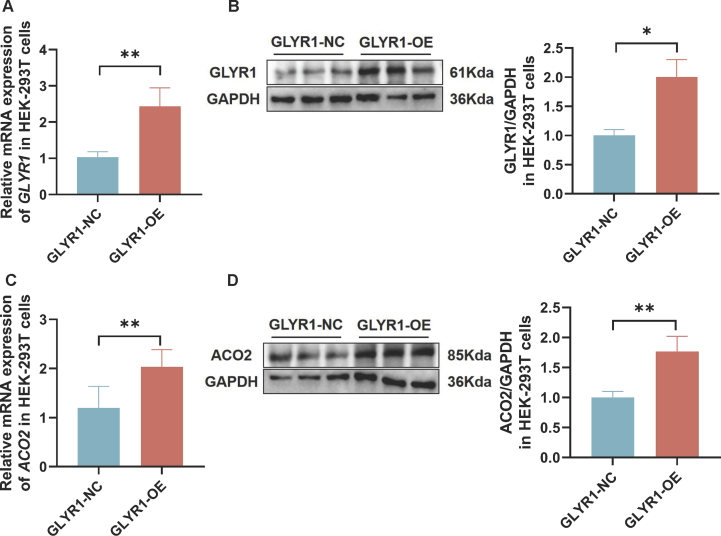


Bioinformatic screening using the Human Transcription Factor Database (HumanTFDB) identified GLYR1 as the top-ranked predicted regulatory factor of ACO2 (Fig. 8A). The predicted binding site of GLYR1 within the ACO2 promoter is shown in Fig. 8B. Analysis via the HumanBase platform further indicated physical interaction between the promoter regions of GLYR1 and ACO2 (Fig. 8C). In the dual-luciferase reporter assay, the luciferase activity in HEK-293T cells co-transfected with the wild-type ACO2 promoter construct and GLYR1 overexpression plasmid was significantly increased (by 42.7 %, p < 0.01) compared to cells transfected only with the wild-type ACO2 plasmid (Fig. 8D). To verify specific binding, EMSA were performed using purified GLYR1 protein and a biotin-labeled DNA probe containing the predicted binding sequence. A clear band shift was observed in the presence of GLYR1, indicating specific protein-DNA complex formation, which was effectively competed by a 100-fold excess of unlabeled probe (Fig. 8E). To investigate the regulatory role of GLYR1 on ACO2 expression, gain-of-function experiments were conducted using a GLYR1 overexpression plasmid (Fig. S6A and B). GLYR1 overexpression significantly increased both ACO2 mRNA and protein contents (Fig. S6C and D).

We next examined whether GLYR1-mediated regulation of ACO2 influences mitochondrial function. Under H/R conditions, GLYR1 overexpression enhanced ATP production and mtDNA copy number while reducing MitoSOX fluorescence. These effects were attenuated by ACO2 knockout, which decreased ATP content, reduced mtDNA copy number, and increased mitochondrial superoxide production (Fig. 8F–J). To assess the impact on apoptosis, we analyzed apoptosis-related gene expression. GLYR1 overexpression under H/R stress upregulated the mRNA expression *Bcl-2* and downregulated *BAX* and *caspase-9*. These changes were reversed by ACO2-KO, which reduced *Bcl-2* expression and elevated *BAX* and *caspase-9* levels (Fig. 8K–M). Consistently, GLYR1 overexpression reduced TUNEL-positive staining, whereas ACO2 knockout enhanced apoptotic signaling (Fig. 8N). These results demonstrated that GLYR1 transcriptionally activated ACO2 expression, thereby preserving mitochondrial function and reducing cellular apoptosis under pathological stress.

## 4 Discussion

Our single-cell transcriptomic profiling identifies endothelial cells as central effectors in LIRI pathogenesis, showing expansion of ECs, monocytes and macrophages consistent with known vascular inflammatory responses to ischemia [23 24]. Concurrent upregulation of endothelial adhesion markers (Cdh5, CD34, VWF, and ICAM4) indicates an activated endothelial phenotype that may promote leukocyte recruitment and vascular leakage [25]. KEGG analysis revealed significant TCA cycle upregulation, highlighting mitochondrial metabolic adaptation as a key stress response mechanism in endothelial cells. The identification of ACO2 as a core TCA cycle-related hub gene underscores its potential importance in LIRI progression. The paradoxical ACO2 expression pattern-elevated in serum but reduced in endothelial cells-likely reflects mitochondrial damage and cellular leakage, positioning serum ACO2 as a potential injury biomarker. Intracellular ACO2 depletion may impair TCA cycle function, contributing to mitochondrial failure and apoptosis, consistent with release patterns of other mitochondrial enzymes in cellular injury.

ACO2 overexpression protects against H/R injury in PVECs by sustaining mitochondrial function. Enhanced ETC gene expression and activity indicate ACO2 maintains oxidative phosphorylation capacity during stress, consistent with mitochondrial enzyme integrity being essential for cellular bioenergetics in ischemia [26]. Reduced oxidative stress and mtDNA damage demonstrate ACO2's additional role in antioxidant defense, supporting findings that aconitase is particularly vulnerable to oxidative inactivation [27]. Notably, ACO2-OE elevated TCA metabolites including cytoprotective itaconate, while increased NAD^+^/NADH and ATP/ADP ratios confirm improved energy efficiency under stress [28 29]. These findings establish ACO2 as a metabolic integrator of TCA cycle flux, ETC function, and redox homeostasis-positioning it as a key regulator of mitochondrial resilience in PVECs.

ACO2 overexpression confers significant protection against I/R-induced lung injury through multimodal mechanisms. Improved gas exchange and attenuated histopathological damage demonstrate preserved pulmonary function and alveolar-capillary integrity [30]. ACO2-OE reduced oxidative stress and inflammation, consistent with mitochondrial metabolites regulating inflammatory cascades [31], while its anti-apoptotic effects via Bcl-2/BAX balance and suppressed cytochrome *c* release indicate mitochondrial apoptosis pathway inhibition [32]. The concordance between in vitro and in vivo findings establishes ACO2-mediated mitochondrial stabilization in PVECs as a key protective mechanism. These results position ACO2 as a promising therapeutic target for LIRI, supported by the clinical feasibility of AAV-mediated lung gene delivery.

Both genetic and pharmacological inhibition of ACO2 consistently exacerbated mitochondrial dysfunction and apoptosis in PVECs under H/R stress, demonstrating its essential role in maintaining endothelial homeostasis. ACO2 deficiency disrupted core mitochondrial functions, including oxidative phosphorylation and redox balance, aligning with the established importance of mitochondrial integrity in ischemic stress [33]. The concordance between knockout and inhibitor models confirms ACO2-specific effects beyond off-target actions. Mechanistically, ACO2 suppression reduced itaconate production, potentially amplifying inflammation through diminished NLRP3 inflammasome regulation. Translationally, ACO2 inhibition worsened pulmonary function, heightened oxidative stress, and enhanced neutrophil-mediated injury in vivo, indicating compromised antioxidant defenses and amplified inflammatory responses. These findings establish ACO2 impairment as a key driver of mitochondrial failure in LIRI, positioning it as a critical node integrating metabolic, oxidative, and inflammatory pathways in I/R injury.

4-OI effectively rescued mitochondrial dysfunction in ACO2-deficient PVECs, establishing itaconate as a crucial downstream mediator of ACO2 protection. 4-OI restored mitochondrial mass, membrane potential and respiratory function, overcoming the metabolic blockade caused by ACO2 deficiency. Beyond metabolic restoration, 4-OI enhanced ETC activity and cellular energy status while exerting anti-apoptotic effects, suggesting broader functions through pathways like Nrf2 activation [34]. The efficacy of 4-OI in rescuing ACO2-KO phenotypes highlights the therapeutic potential of targeting the ACO2-itaconate axis. As a cell-permeable derivative, 4-OI offers clinical advantages while maintaining biological activity. These findings establish the ACO2-itaconate pathway as a promising target for treating LIRI and other mitochondrial disorders.

This study identifies GLYR1 as a novel transcriptional regulator of ACO2 that maintains mitochondrial function under stress. Bioinformatic prediction and experimental validation confirmed GLYR1's direct binding to the ACO2 promoter, with reduced luciferase activity suggesting potential repressor function. GLYR1 overexpression enhanced mitochondrial parameters through ACO2-dependent mechanisms, as ACO2 knockout abolished these protective effects. The GLYR1-ACO2 axis also mediated anti-apoptotic responses, with GLYR1 overexpression rebalancing Bcl-2/BAX ratios and suppressing caspase activation in an ACO2-dependent manner. These findings establish GLYR1 as an upstream regulator of the mitochondrial protection pathway, offering new therapeutic targets for LIRI and related disorders.

While this study provides comprehensive evidence supporting the central role of ACO2 in LIRI pathogenesis, several limitations should be acknowledged. First, the therapeutic potential of 4-OI, though promising, was evaluated in a relatively short-term intervention paradigm; its long-term efficacy and potential side effects warrant additional investigation. Secondly, while we observed a correlation between serum ACO2 contents and disease severity, larger prospective clinical studies are needed to establish its definitive diagnostic and prognostic value in human LIRI.

## CRediT authorship contribution statement

**Jiaojiao Sun:** Data curation, Formal analysis, Funding acquisition, Investigation, Validation, Writing – review & editing. **Bo Xu:** Investigation, Validation. **Yijing Chen:** Writing – review & editing. **Meng Sui:** Supervision, Writing – review & editing. **Mochi Wang:** Investigation. **Ranming Ma:** Investigation. **Jinbo Wu:** Investigation, Validation. **Shiyong Teng:** Investigation, Validation. **Qingfeng Pang:** Data curation, Formal analysis, Funding acquisition, Investigation, Validation, Visualization, Writing – original draft. **Chunxiao Hu:** Conceptualization, Funding acquisition, Methodology, Project administration, Resources, Software, Supervision, Writing – original draft, Writing – review & editing.

## Declaration of competing interest

The authors declare that they have no known competing financial interests or personal relationships that could have appeared to influence the work reported in this paper.

## Figures and Tables

**Fig:**
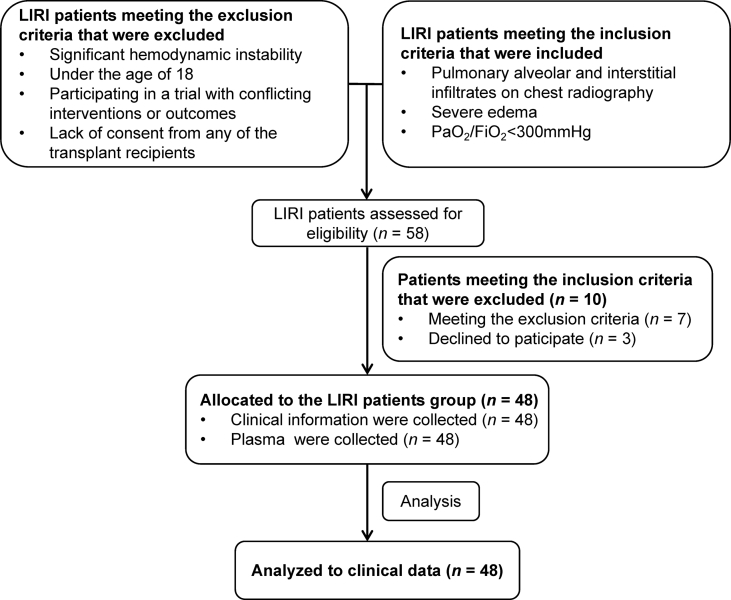
S1

**Fig:**
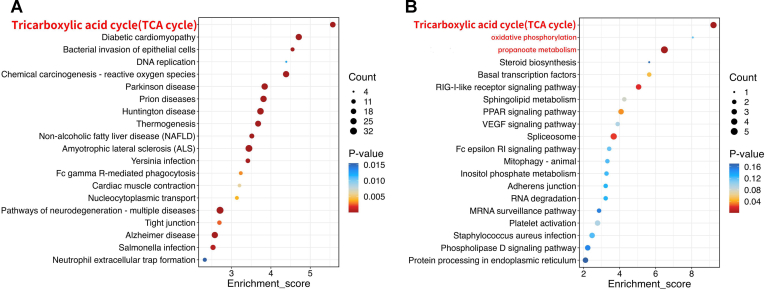
S2

## Data Availability

Data will be made available on request.

## Abbreviations:

LIRI, Lung ischemia-reperfusion injury; PVECs, Pulmonary vascular endothelial cells; ACO2, Aconitase-2; TCA, PaO_2_, partial pressure of oxygen; PaCO_2_, Partial Pressure of Carbon Dioxide; Tricarboxylic acid; TA, Tricarballylic acid; 4-OI, 4-octyl itaconate; BALF, Bronchoalveolar lavage fluid; H/R, Hypoxia/Reoxygenation; MDA, Malondialdehyde; MPO, Myeloperoxidase, GSH, Glutathione; SOD, Superoxide dismutase; T-AOC, Total Antioxidant Capacity; Cyt-c, Cytochrome C; ATP, Adenosine triphosphate content; ETC, Electron Transport Chain; OCR, Oxygen Consumption Rate; ACO2-KO, ACO2-knockout; ACO2-OE, ACO2 overexpression.
