# Supplementary material for: Unveiling a novel function of Aconitase-2: attenuating lung ischemia-reperfusion injury via inhibition of pulmonary endothelial apoptosis
Source: Redox Biol. 2026 Jan 12;90:104016. doi: 10.1016/j.redox.2026.104016 (PMC12891905; doi:10.1016/j.redox.2026.104016)
Supplement: Multimedia component 1 [file mmc1.docx]

**Supplemental Figure Legends**

**Fig. S1. Trial flow diagram of plasma collected from patients with healthy**

**donors and LIRI patients.**

**Fig. S2. Single-cell and transcriptomic profiling I/R mice.** (A) KEGG analysis of scRNA-seq. (B) KEGG analysis of RNA-seq.

**Fig. S3. ACO2 inhibition exacerbated mitochondrial damage and apoptosis in primary PVECs.** (A) Cell viability assessed by CCK-8 assay following ACO2 inhibitor treatment. (B) Viability of primary PVECs exposed to H/R injury. (C) Dose-response evaluation of ACO2 inhibitor efficacy by CCK-8. (D-E) ACO2 expression levels measured by RT-qPCR and western blot. (F) Mitochondrial ultrastructure examined by TEM. The red arrows indicate the disappearance of mitochondrial cristae. (G) Cellular ATP content determined by enzymatic assay. (H) Mitochondrial superoxide production detected using MitoSOX Red (scale bar = 20 μm). (I) Cyt-c release from mitochondria. (J) Apoptosis-related gene expression (*BAX, Bcl-2,* and *caspase-9*) analyzed by RT-qPCR. (K) Apoptotic cells visualized by TUNEL staining (scale bar = 20 µm). Data are presented as the mean ± SD; *n* = 3; **P* < 0.05, ***P* < 0.01, ****P* < 0.001, *****P* < 0.0001, and ns indicates no significant difference.

**Fig. S4.** **ACO2 inhibition exacerbated mitochondrial damage and apoptosis in I/R mice.** (A) Schematic of TA administration in I/R mice. (B-C) *ACO2* expression levels assessed by RT-qPCR and western blot. (D) Blood gas parameters (PaO_2_ and PaCO_2_) following I/R injury. (E) Representative H&E-stained lung sections (scale bars = 20/50 μm, Black arrow: Alveolar expansion; Red arrow: Hemorrhage; Green arrow: Alveolar septum thickening and inflammatory cell infiltration). (F) Quantitative assessment of lung injury via histopathological scoring. (G) Total cells count and protein concentration in BALF. (H-K) Oxidative stress parameters in lung tissue: GSH, SOD, MDA, and MPO. (L) MPO immunohistochemical staining with semiquantitative analysis (scale bar = 20 µm, Red arrow: MPO positive; Green arrow: MPO negative). Data are presented as the mean ± SD; *n* = 6; **P* < 0.05, ***P* < 0.01, ****P* < 0.001, and ns indicates no significant difference.

**Fig. S5. 4-OI rescued mitochondrial dysfunction in primary PVECs.** (A) Mitochondrial mass assessed by MitoTracker staining (scale bar = 20 µm). (B) Mitochondrial respiratory function measured by OCR. (C) Mitochondrial membrane potential evaluated using JC-1 staining (scale bar = 20 µm). (D) Activities of mitochondrial ETC complexes. (E) Protein contents of ETC complex subunits. (F) Cyt-c release from mitochondria. (G) Apoptosis-related protein contents (cleaved caspase-3, Bcl-2, and BAX) analyzed by western blot with semi-quantitative analysis. Data are presented as the mean ± SD; *n* = 3; **P* < 0.05, ***P* < 0.01, and ****P* < 0.001.

**Fig. S6. GLYR1 overexpression increased ACO2 mRNA and protein contents in HEK-293T cells.** (A) *GLYR1* mRNA expression analyzed by RT-qPCR. (B) GLYR1 protein contents assessed by western blot with semi-quantitative analysis. (C) *ACO2* mRNA expression measured by RT-qPCR. (D) ACO2 protein contents were determined by western blot and semi-quantification. Data are presented as the mean ± SD; *n* = 3; **P* < 0.05, and ***P* < 0.01.
