# Supplementary material for: Unveiling a novel function of Aconitase-2: attenuating lung ischemia-reperfusion injury via inhibition of pulmonary endothelial apoptosis
Source: Redox Biol. 2026 Jan 12;90:104016. doi: 10.1016/j.redox.2026.104016 (PMC12891905; doi:10.1016/j.redox.2026.104016)
Supplement: Multimedia component 2 [file mmc2.docx]

**Table S1 The characteristics of healthy donors and LIRI patients**

|  | **Heathy donors (*n* = 65)** | **LIRI patients (*n* = 48)** | **Inspection value**  **(*t*, or *χ*^2^)** | ***P* value** |
| --- | --- | --- | --- | --- |
| Age (range) | 75.4 ± 5.5 (47 - 98) | 74.1 ± 8.6 (55 - 91) | 1.456 | 0.105 |
| Sex (%) | Male = 38 (58.5%) | Male = 22 (45.8%) | 0.287 | 0.696 |
| BMI (kg/m^2^) | 21.5 ± 3.8 | 26.2 ± 2.2 | 1.192 | 0.321 |
| Smoking status | Smoker = 39 (60.0%) | Smoker = 28 (58.3%) | 0.667 | 0.222 |
| Hypertension | 33 (50.8%) | 26 (54.2%) | 1.021 | 0.088 |
| COPD | 39 (60.0%) | 29 (60.4%) | 0.673 | 0.162 |
| PaO_2_ | 156.7 ± 90.7 (39.6 - 267.5) | 76.8 ± 56.4 (23.5 - 167.7) | 1.231 | 0.001 |
| PaCO_2_ | 47.5 ± 38.4 (8.5 - 96.6) | 67.6 ± 50.5 (8.6 - 170.5) | 0.786 | 0.023 |
| PaO_2_/FiO_2_ (mmHg) | 754.5 ± 546.5 (56.4 - 1586.5) | 253.3 ± 215.5 (18.4 - 557.5) | 0.467 | 0.001 |
| PCT [ng/mL; IQR] | 0.3 [0.2, 1.1] | 5.3 [1.2, 8.4] | 0.134 | 0.087 |
| CRP (mg/L; range) | 68.5 ± 51.8 (8.1 - 151.6) | 105.9 ± 87.9 (13.7 - 235.9) | 0.763 | 0.077 |

Variables were presented as mean ± SD, number of patients (*n*) or median [IQR]. COPD, chronic obstructive pulmonary disease; PaO_2_, partial pressure of oxygen; PaCO_2_, Partial Pressure of Carbon Dioxide; PCT, procalcitonin; CRP, C-reactive protein; IQR, interquartile range.
