# Supplementary material for: Unveiling a novel function of Aconitase-2: attenuating lung ischemia-reperfusion injury via inhibition of pulmonary endothelial apoptosis
Source: Redox Biol. 2026 Jan 12;90:104016. doi: 10.1016/j.redox.2026.104016 (PMC12891905; doi:10.1016/j.redox.2026.104016)
Supplement: Multimedia component 3 [file mmc3.docx]

# Table S2. The antibodies used in the present study

| **Antibody** | **Source** | **Host** | **Dilution** |
| --- | --- | --- | --- |
| ACO2 | 6922, Cell Signaling Technology | rabbit | 1:1000 |
| NDUF9B | 99235, Cell Signaling Technology | rabbit | 1:1000 |
| NDUFV1 | 11238-1-AP, proteintech | rabbit | 1:1000 |
| SDHA | 5839, Cell Signaling Technology | rabbit | 1:1000 |
| SDHB | 92649, Cell Signaling Technology | rabbit | 1:1000 |
| CYC1 | 10242-1-AP, proteintech | rabbit | 1:1000 |
| COX4 | 4850, Cell Signaling Technology | rabbit | 1:1000 |
| ATP5a1 | 18023, Cell Signaling Technology | rabbit | 1:1000 |
| Cleaved Caspase-3 | 9664, Cell Signaling Technology | rabbit | 1:1000 |
| pro-caspase3 | ab32150, Abcam | rabbit | 1:1000 |
| Bcl2 | 15071, Cell Signaling Technology | mouse | 1:1000 |
| BAX | 2772, Cell Signaling Technology | rabbit | 1:1000 |
| GLYR1 | 14833-1-AP, proteintech | rabbit | 1:1000 |
| MPO | 66177-1-Ig, proteintech | mouse | 1:400 |
| GAPDH | 5174, Cell Signaling Technology | rabbit | 1:1000 |
