# Supplementary material for: Unveiling a novel function of Aconitase-2: attenuating lung ischemia-reperfusion injury via inhibition of pulmonary endothelial apoptosis
Source: Redox Biol. 2026 Jan 12;90:104016. doi: 10.1016/j.redox.2026.104016 (PMC12891905; doi:10.1016/j.redox.2026.104016)
Supplement: Multimedia component 4 [file mmc4.docx]

**Table S3. Primer sequences of the target genes**

| **Target Gene** | **Primer** | **Primer Sequences (5’ to 3’)** |
| --- | --- | --- |
| *GAPDH (mouse)* | Forward  Reverse | AGGTCGGTGTGAACGGATTTG  TGTAGACCATGTAGTTGAGGTCA |
| *ACO2 (mouse)* | Forward  Reverse | ATACCGCCATCTTCA  ATCCTCCTTAGTCCTTT |
| *Ndufa6 (mouse)* | Forward  Reverse | TCAGCGACGGAAAGAGTATGA  CCACTGGTTTCTGACTGGATGT |
| *Ndufb9 (mouse)* | Forward  Reverse | GTGGTGCGTCCAGAGAGAC  GGCCTTCGCCATATCCTTTTC |
| *Ndufv1 (mouse)* | Forward  Reverse | AGGATGAAGACCGGATTTTCAC  CAGTCACCTCGACTCAGGGA |
| *Sdha (mouse)* | Forward  Reverse | CAAACAGGAACCCGAGGTTTT  CAGCTTGGTAACACATGCTGTAT |
| *Sdhb (mouse)* | Forward  Reverse | ACAGCTCCCCGTATCAAGAAA  GCATGATCTTCGGAAGGTCAA |
| *Cyc1 (mouse)* | Forward  Reverse | CTTCGCGGGGTAGTGTTGG  GGCCAGACTTCGACGACAA |
| *Uqcrc1 (mouse)* | Forward  Reverse | ATACCGCCATCTTCA  ATCCTCCTTAGTCCTTT |
| *Uqcrc2 (mouse)* | Forward  Reverse | ACCTGGCGGTGCTTCATATC  CGTGGGTACTTGCGCTTACT |
| *Cox4i1 (mouse)* | Forward  Reverse | TTGGCAAGAGAGCCATTTCT  GCGTAAGTGGGGAAAGCATA |
| *Atp5a1 (mouse)* | Forward  Reverse | AGCCTCGGTAATGCCCTA  GCTTTGCGGTAAGGGTATTGA |
| *Atp5o (mouse)* | Forward  Reverse | GCCCTCGGTAATGCTATTGA  GCCCTCGGTAATGCTATTGA |
| *mt-Nd1 (mouse)* | Forward  Reverse | TCCGAGCATCTTATCCACGC  GTATGGTGGTACTCCCGCTG |
| *mt-Co1(mouse)* | Forward  Reverse | CTACCCACCTCTAGCCGGAA  TGTTATGGCTGGGGGTTTCA |
| *mt-Co2 (mouse)* | Forward  Reverse | ACCGAGTCGTTCTGCCAATA  ATTTAGTCGGCCTGGGATGG |
| *mt-Atp6 (mouse)* | Forward  Reverse | ACGCCTAATCAACAACCGTC  TTCGTCCTTTTGGTGTGTGGA |
| *Bcl2 (mouse)* | Forward  Reverse | TTGAGCATAGTGGTTGGAC  TGGAGGAGGAGAGGAGAA |
| *BAX (mouse)* | Forward  Reverse | ACGCTGTCCAAACCGTC  TTAACCAGGTCAAGAGCAC |
| *Caspase 9 (mouse)* | Forward  Reverse | CTCTTGAAATAGCAGACGGG  CAGGTGATAACTAAAACGGTCA |
